# Development of Porous Polyurethane Implants Manufactured via Hot-Melt Extrusion

**DOI:** 10.3390/polym12122950

**Published:** 2020-12-10

**Authors:** Ioannis Koutsamanis, Martin Spoerk, Florian Arbeiter, Simone Eder, Eva Roblegg

**Affiliations:** 1 Research Center Pharmaceutical Engineering GmbH, Inffeldgasse 13, 8010 Graz, Austria; ioannis.koutsamanis@rcpe.at (I.K.); martin.spoerk@rcpe.at (M.S.); simone.eder@rcpe.at (S.E.); 2 Institute of Pharmaceutical Sciences, Pharmaceutical Technology and Biopharmacy, University of Graz, Universitaetsplatz 1, 8010 Graz, Austria; 3 Institute of Materials Science and Testing of Polymers, Montanuniversitaet Leoben, Otto Gloeckel-Straße 2, 8700 Leoben, Austria; Florian.Arbeiter@unileoben.ac.at

**Keywords:** extrusion, pore former, scaffold, thermoplastic polyurethane, polymer, foam

## Abstract

Implantable drug delivery systems (IDDSs) offer good patient compliance and allow the controlled delivery of drugs over prolonged times. However, their application is limited due to the scarce material selection and the limited technological possibilities to achieve extended drug release. Porous structures are an alternative strategy that can overcome these shortcomings. The present work focuses on the development of porous IDDS based on hydrophilic (HPL) and hydrophobic (HPB) polyurethanes and chemical pore formers (PFs) manufactured by hot-melt extrusion. Different PF types and concentrations were investigated to gain a sound understanding in terms of extrudate density, porosity, compressive behavior, pore morphology and liquid uptake. Based on the rheological analyses, a stable extrusion process guaranteed porosities of up to 40% using NaHCO_3_ as PF. The average pore diameter was between 140 and 600 µm and was indirectly proportional to the concentration of PF. The liquid uptake of HPB was determined by the open pores, while for HPL both open and closed pores influenced the uptake. In summary, through the rational selection of the polymer type, the PF type and concentration, porous carrier systems can be produced continuously via extrusion, whose properties can be adapted to the respective application site.

## 1. Introduction

Over the last 20 years, the pharmaceutical industry has shown an ever-growing interest in hot-melt extrusion (HME) as a manufacturing technique [1,2]. What has been a golden standard in the polymer industry since the middle of the 20th century [3] has become a topic of rigorous research for the development of novel drug delivery systems [4]. In the pharmaceutical industry, HME owes its popularity to several inherent advantages. Examples are the solvent-less (green) and continuous nature of the technique [5], the increasing drug bioavailability [6] and the utilization in the development of different types of drug-delivery systems. So far, HME has been investigated for the manufacturing of pellets [7,8], tablets [9], gastro retentive systems [10], nanopharmaceuticals [11,12], protein delivery carriers [13,14,15] and shaped drug-delivery systems intended for topical and parenteral applications, such as vaginal films [16], intra-vaginal rings [17,18,19,20] and implantable drug-delivery systems (IDDS) [21,22,23,24,25]. Thereby, advancements in controlled-release systems have brought IDDS into the center of extensive research for several decades [26,27,28,29,30].

HME-based IDDS can be classified into biodegradable and non-biodegradable [29,30]. Biodegradable IDDS offer the advantage that they do not need to be removed after treatment. However, the inert nature of non-biodegradable IDDS is also beneficial in certain aspects. Among the advantages are longer drug-release profiles [29], less complicated drug release mechanisms due to fewer influencing factors [31,32], outstanding physicochemical stability and lower costs [27,33]., Although all IDDS must fulfill high regulatory requirements, non-biodegradable materials do not require the additional ISO biological evaluations, i.e., identification, evaluation and toxicokinetic studies of degradation products [27,33]. However, when designing a non-biodegradable IDDS, the mechanical properties of the material must be taken into account, as a flawless insertion and removal from the body without material failure must be guaranteed [34]. This requires the use of materials that have a certain degree of flexibility and damage tolerance [35,36]. Of the whole range of polymer candidates suitable for HME [6,37], only a few are flexible enough to be used for the manufacturing of non-biodegradable IDDS [28]. One example is poly(ethylene-vinyl acetate) (EVA), a non-biodegradable copolymer of ethylene and vinyl acetate. Compared to pure polyethylene, EVA is preferred for drug delivery applications due to its higher drug permeability, which in turn is a function of the varying (up to 40 wt%) vinyl acetate content in the copolymer [18,38,39,40]. Therefore, besides commercialized IDDS (e.g., Probuphine^®^, Nexplanon^®^, Iluvien^®^ and Retisert^®^), EVA has been implemented for oral applications [41,42], drug-releasing stents [43] and vaginal rings (Nuvaring^®^, generics thereof and rings in development [18,19,44,45,46]). However, owing to the polymer’s hydrophobic nature, EVA is mainly used for the delivery of hydrophobic small molecules [17,47].

An alternative to EVA is thermoplastic polyurethanes (TPUs). TPUs consist of two thermodynamically incompatible segments, namely hard and soft segments. Hard segments comprise urethanes (isocyanate with a low molecular weight chain extender), while soft segments consist of polyols (usually polyesters or polyethers) [48,49]. This structure reveals unique and versatile physicochemical properties. In contrast to EVA, TPUs can be hydrophilic or hydrophobic depending on the chemical composition of their soft segments [48,50]. Hydrophilic TPUs tend to swell upon contact with water without degrading or dissolving, which impacts drug release kinetics [48]. This flexibility in terms of hydrophobicity and hydrophilicity renders TPUs as materials of choice for the delivery of a wide range of active pharmaceutical ingredients (APIs). Similar to EVAs, hydrophobic small molecules show adequate permeability in hydrophobic TPUs, and the drug release is achieved mainly via solute diffusion [32,48]. In contrast to EVAs, hydrophilic molecules can be delivered from hydrophilic TPUs via a combination of solute diffusion, swelling and liquid uptake capacities of the polymer matrix [51,52]. Additionally, by altering the ratio of soft and hard segments, a wide selection of TPUs with different mechanical properties, mostly represented by varying hardness values, are available depending on the desired application. A broad mechanical portfolio is beneficial for employing TPUs as IDDS, as not only the biomechanical properties of implants need to be as similar as possible to the properties of the adjacent tissue [36,53], but also the drug release rates can be influenced by the ratio of soft to hard segments [54]. Recent studies have confirmed the great potential of non-biodegradable TPUs to deliver various APIs from IDDS [54,55,56] and vaginal rings [57,58,59,60] but also as matrix excipients in oral tablets [49,61,62].

In addition to the changes in physicochemical properties, several other strategies can be implemented to tailor the drug release and mechanical properties of TPU carriers. One promising technique for fine-tuning drug release is the introduction of porous structures. So far, polyurethane scaffolds and porous systems have been investigated in the field of regenerative medicine [63,64,65] and as drug delivery systems [66,67,68] using, e.g., the solvent-casting particulate leaching method. In none of these studies, however, pores were continuously formed via HME, although HME is already well established for the implementation of porous structures in the polymer industry [69,70,71,72]. In the pharmaceutical industry only a handful of studies has evaluated the applicability of foaming during HME [10,73,74,75]. However, none of the used materials are non-biodegradable. Only recently, Claeys et al. [61,62] have used water-soluble additives to create porous structures in contact with water and to modify the drug release from TPU-based tablets produced via HME. This strategy could be relevant for IDDS applied at sites with sufficient liquid to initiate the formation of pores. Since IDDS are often implanted in regions with limited amount of body fluid, e.g., subcutaneously, pores must already exist prior to administration. However, such systems, where pores are introduced during a continuous process in a controlled way have not yet been systematically studied.

The present study aims at closing this gap by presenting an in-situ technique for creating porous TPU-based IDDS via a one-step HME process using chemical pore formers. As a decisive first step toward a versatile platform for novel IDDS, the structural properties of the systems were thoroughly investigated with regard to their porosity, pore morphology, mechanical properties and liquid uptake kinetics. By skillful selection of the amount and type of pore formers and TPUs, optimal material and processing parameters were determined to achieve reproducible porous structures.

## 2. Materials and Methods

### 2.1. Materials

The hydrophilic TPU Pathway^®^ PT83AE100 (HPL, shore hardness = 83 A, nominal water uptake = 100 wt%) and the hydrophobic TPU Pathway^®^ PT72AE (HPB, shore hardness = 72 A) were kindly provided by Lubrizol LifeSciences, Wickliffe, OH, USA. Ammonium bicarbonate (NH_4_HCO_3_) and sodium bicarbonate (NaHCO_3_), supplied by Sigma Aldrich, Vienna, Austria, were used as pore formers (PFs). Sodium hydroxide, potassium dihydrogen phosphate (all by Sigma Aldrich, Vienna, Austria) and water purified by TKA MicroPure UV (JWT GmbH, Jena, Germany) were used to prepare the aqueous media.

### 2.2. Methods

#### 2.2.1. Rheological Properties and Thermal Characterization

Rheology tests were performed to assess the processing temperature of the TPUs. The respective specimens were prepared by vacuum compression molding (VCM, MeltPrep GmbH, Graz, Austria). Per specimen, 700 mg of polymer were placed in the VCM sample chamber, which was heated to 170 °C for 7 min followed by cooling to ambient temperature under constant vacuum (10^−3^ bar). The samples of 1.2 mm in thickness and 25 mm in diameter were analyzed using the rheometer Physica MCR 301 (Anton Paar GmbH, Graz, Austria) in plate–plate geometry at a gap of 1 mm under nitrogen. The viscosity of each polymer was measured in an oscillatory shear-controlled mode in its linear viscoelastic region at 160, 170 and 180 °C between 0.1 and 628 s^−1^ (*n* = 3).

To evaluate the thermal decomposition temperature range of the two PFs, differential scanning calorimetry (DSC) was performed using a 204 F1 Phoenix apparatus (Netzsch GmbH and Co. KG, Selb, Germany) equipped with an external cooling system and an automated sampling unit. Three samples per PF, each with a mass of 7 ± 1 mg of each PF, were exposed to a single heating cycle from 25 to 200 °C with a heating rate of 10 °C·min^−1^ under a constant nitrogen flow of 50 mL·min^−1^. The obtained thermograms were analyzed with the Netzsch Proteus analysis software.

#### 2.2.2. Manufacturing of the Porous Extrudates

To ensure a homogeneous distribution of the PF within the TPUs, the polymers were cryo-milled prior to processing (SPEX 6875 SamplePrep, Metuchen, NJ, USA) and dried according to the provider’s datasheets [76]. As a result of the rheological and thermal characterization (see Section 3.1), the cryo-milled TPUs were blended with 1, 3 and 5 wt% of NaHCO_3_ in a 3D shaker mixer at 60 Hz for 20 min (TURBULA^®^ T2F, Willy A. Bachofen AG, Muttenz, Switzerland). Of pure TPUs and TPU/PF blends 50 g of each were manually fed into the lab-scale extruder ZE 9 HMI (Three-Tec GmbH, Seon, Switzerland) equipped with two corotating screws (9 mm in diameter), an L/D ratio of 20 and a die of 4 mm in diameter. The extrusions were performed at a screw speed of 40 rpm and at 160 °C (HPB) or 170 °C (HPL). The extrudates were cooled by natural convection, manually cut into segments of 10 cm and stored in the temperature-controlled cabinet KBF720 (Binder GmbH, Tuttlingen, Germany) at 25 °C/60% RH prior to subsequent characterizations.

#### 2.2.3. Apparent Density and Ovality Measurements

The apparent (geometric) density was calculated from the mass (Entris 224I analytical balance, Sartorius AG, Göttingen, Germany), the length (caliper) and diameter of each extrudate segment as

(1) $$Apparent\;density\;\left(\rho \right)=\frac{m}{\pi \cdot R^{2}\cdot L}$$

in which *m* (g), *R* (cm) and *L* (cm) refer to the mass, the cross-sectional radius and the length of the extrudate segment, respectively.

The diameter and ovality, i.e., the minimum diameter subtracted from the maximum diameter, were measured offline using the ODAC^®^ laser gauge paired with the USYS 2000 data acquisition and display system (Zumbach Electronic AG, Orpund, Switzerland). The mean of at least 20 measured diameters per segment (*n* = 15 segments per formulation), taking into consideration the ovality, was used for the calculation. All obtained apparent densities were evaluated to a significance level of 5% (Student’s *t*-test, assuming unequal variance, two-tailed, with *p* < 0.05 being considered as significant).

#### 2.2.4. True and Skeletal Density

The cryo-milled polymers’ true density and extrudates’ skeletal densities were determined by means of helium pycnometry (AccuPyc II 1340, Micromeritics Instrument Corp., Norcross, GA, USA). The volume of the manually cut extrudates was measured using 20 gas purges at 19.5 psi with an equilibrium rate of 0.005 psi·min^−1^ until five consecutive volume values with a relative standard deviation < 1% were obtained (*n* = 3 for each formulation). The true/skeletal density was calculated as the ratio of the sample mass and calculated volume. The percentage of open pores was calculated based on Ref. [77] as

(2) $$Open\;Pores\;\left(\%\right)=\frac{\nu_{apparent}-\nu_{skeletal}}{\nu_{apparent}}\times 100$$

in which *v_apparent_* refers to the specific volume (reciprocal apparent (geometric) density = 1/ρ) and *v_skeletal_* refers to the skeletal specific volume (reciprocal skeletal density). The percentage of closed pores was calculated by comparing the skeletal specific volume of the porous extrudates and the true specific volume of the cryo-milled polymers (*v_true_*):

(3) $$Closed\;Pores\;\left(\%\right)=\frac{\nu_{skeletal}-\nu_{true}}{\nu_{skeletal}}\times 100$$

The total porosity was calculated from the sum of open (Equation (2)) and closed pores (Equation (3)). All obtained values were evaluated to a significance level of 5% by considering the error propagation.

#### 2.2.5. Pore Morphology

For the analysis of the pore size and shape, at least 20 samples of each extrudate were analyzed via bright-field microscopy (BX51M, Olympus, Hamburg, Germany). Extrudate segments were cryo-cut in the manual microtome MT.5503 (Euromex, Arnhem, The Netherlands) to a thickness of approximately 300 µm after immersion into liquid nitrogen for 30 s. Images of each sample were recorded at a magnification of 40× (*n* = 75 images per formulation). The pore areas on planar cuts were calculated in the software ImageJ (open source), as in Ref. [78], and reported in the form of histograms, as recommended by [79]. To improve the visibility, the pore morphology of cryo-fractured and gold sputtered extrudates were additionally investigated by means of scanning electron microscopy (SEM) on a Tescan Vega II (Tescan Brno s.r.o., Brno, Czech Republic) at 20 kV using secondary electrons.

#### 2.2.6. Mechanical Properties

The compression tests were performed on small cylindrical specimens (5 mm length) directly cut from the produced filaments. The specimens were mounted between two compression plates on a Zwick Z010 universal testing machine (ZwickRoell GmbH and Co. KG, Ulm, Germany) and deformed with a constant crosshead speed of 10 mm·min^−1^. Compression forces were measured with a 10 kN load cell. Strain values were calculated from crosshead displacement during the test, since the specimen height did not allow the use of a contact extensometer. To account for machine stiffness, blind curves without specimens were performed and subtracted from the measured curves. The compression modulus (EC) evaluated between 0.05% and 0.25% strain and compressive stress at a 50% compressive strain (σ_50_) based on five repetitions were evaluated to a significance level of 5%.

#### 2.2.7. Liquid Uptake

To determine the liquid uptake of the systems, segments (10 ± 0 cm length and 1.7 ± 0.3 g mass) of each formulation (*n* = 3) were manually cut with a razor blade, weighed using an analytical balance (Entris 224I analytical balance, Sartorius AG, Göttingen, Germany), and immersed into 100 mL of USP phosphate buffer (pH 7.4), at 37 °C, in an incubator shaker operated at 130 rpm. The buffer system was chosen due to its similarity regarding pH and osmolarity with blood and other physiological body fluids. Metal paperclips were used to prevent the samples from floating. The samples were removed after 15 and 30 min, 1, 2, 4, 8, 12 24, 48, 72, 144 and 216 h, gently blotted to remove the excess of medium from the samples’ surface and weighed again. The liquid uptake was calculated using the following equation:

(4) $$Liquid\;Uptake\;\left(\%\right)=100\times \frac{Sample\;Mass\;Wet\;\left(g\right)-Sample\;Mass\;Dry\;\left(g\right)}{Sample\;Mass\;Dry\;\left(g\right)}$$

## 3. Results and Discussion

### 3.1. Rheological Properties, Thermal Characterization and Manufacturing

**Figure 1:**
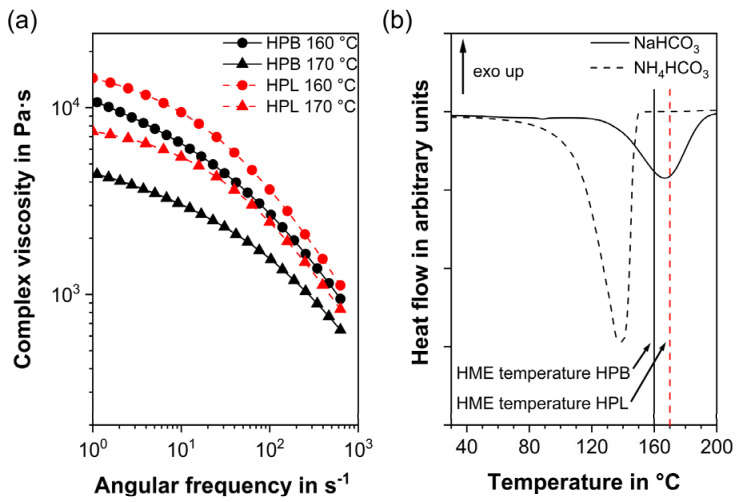
(**a**) Mean viscosity curves of three individual measurements for the hydrophobic (HPB) and hydrophilic thermoplastic polyurethane (HPL) at their recommended hot-melt extrusion temperatures and (**b**) differential scanning calorimetry thermograms of the pore formers with the characteristic endothermic decomposition peaks of the carbonate-based pore formers.

As expected from the shear-thinning behavior of all TPUs [60,80], the melt viscosity decreased with increasing temperature and angular frequency for both TPUs investigated (Figure 1). At the same temperature, HPB revealed a significantly lower viscosity than HPL for all angular frequencies. This finding can be attributed to the polymers’ different fraction of hard segments. Although both polymers comprise the same type of hard segments (aliphatic hexamethylene diisocyanate and 1,4-butanediol) they differed in the number of hard segments. Compared to HPB, which revealed a soft to hard segment ratio of 12.1, HPL consisted of more hard segments (soft to hard segment ratio of 11.8) [60]. Such a higher fraction of hard segments inhibited the molecular movements of the soft segments [62]. Consequently, TPUs consisting of more hard segments (HPL) revealed a higher chain rigidity [81] and therefore higher shore hardness (83 A for HPL compared to 72 A for HPB) and elevated melt viscosity compared to HPB [62,82]. As stated by Hiltz, the correlation between soft/hard segments ratio and melt viscosity has been attributed to increased phase mixing between the segments, which consequently increases the T_g_ of the polymer [83], while, according to Claeys et al., the increase in rigidity is a function of the heat capacity at the T_g_ of the polymer [62].

The melt viscosity of HPB at 160 °C was very similar to that of HPL at 170 °C, particularly at higher angular frequencies that reflected the shear condition in a twin-screw extruder. Only at these temperatures, the polymers also show a complex viscosity between 10^3^ and 10^4^ Pa·s, which is recommended for adequate mixing and to avoid exceeding torque limits during the HME process [84]. Therefore, and in order to process both polymers at comparable rheological conditions, 160 and 170 °C were chosen as the processing temperatures for HPB and HPL, respectively. To avoid further influencing parameters, the screw speed was kept constant at 40 rpm. Additional adaptations of the HME process, such as the screw configuration, screw fill level and feed rate and their effect on porous extrudates will be the subject of subsequent studies.

The choice of the appropriate PF depends mainly on the correlation between the decomposition temperature of the PFs and the process temperature of the polymers and on the compatibility of the polymer with the products of the PF thermal degradation [69,71,85]. PFs that react at higher temperatures than the process temperature result in unreacted, solid PF embedded in the polymer without producing gas and therefore pores [69]. PFs that react at temperatures lower than the process temperature lead to pore forming gases that escape from the feeding port before the polymer melts [69]. This phenomenon occurred with NH_4_HCO_3_, whose decomposition reaction took place between 60 and 150 °C, i.e., at least 10–20 °C below the process temperature recommended for both TPU types (Figure 1b). As a result, the addition of NH_4_HCO_3_ to HPB or HPL resulted in non-porous or inhomogeneous partially porous extrudates, regardless of the PF concentration (Supplementary Information, Figure S1). Porous systems can be introduced by HME only if the processing temperature overlaps with the PF decomposition range [69]. This requirement was only met for NaHCO_3_, whose decomposition temperature was between 120 and 190 °C (Figure 1b), which is in agreement with the literature [86]. In the thermogram of NaHCO_3_, it can be seen that the degradation of the PF was completed within approximately 6 min (endothermic peak onset to end at the 10 K/min heating rate) since the second heating cycle showed no additional degradation. HME was performed at temperatures at the peak range or higher than the degradation peak of NaHCO_3_ and the mean residence time in the extruder was longer than 6 min. Therefore, it was assumed that the low concentrations of NaHCO_3_ used in the formulations reacted completely. Thus, for all subsequent investigations, NaHCO_3_ was chosen as the appropriate PF. Table 1 provides an overview of the final formulations. It should be mentioned, that the thermal degradation of NaHCO_3_ resulted in Na_2_CO_3_ as residue in the porous extrudate. It is known that the compatibility of residues not only with the polymer carrier but also with regard to the application site (i.e., surrounding tissue) must not be neglected [33].

### 3.2. Apparent Density and Ovality Measurements

**Figure 2:**
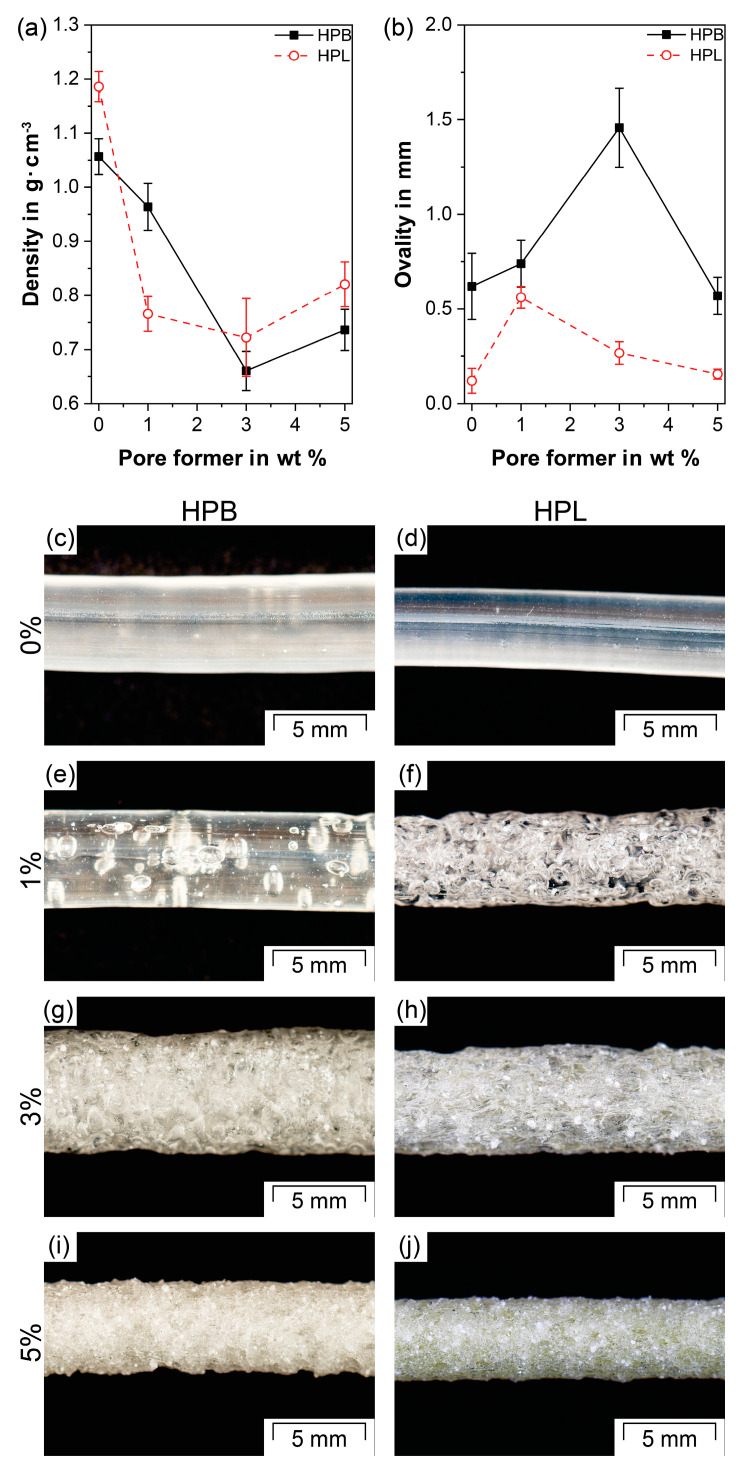
(**a**) Apparent density, (**b**) ovality and (**c**–**j**) macroscopic images of formulations comprising hydrophobic (HPB) and hydrophilic thermoplastic polyurethane (HPL) with and without NaHCO_3_ as the pore former.

Figure 2 shows the apparent density, ovality and macroscopic images of extrudates comprising either HPB or HPL without and with NaHCO_3_ as PF. Compared to the pure TPUs, the addition of NaHCO_3_ resulted in a significant reduction in density at all observed PF concentrations for both HPB and HPL (*p* < 0.05; Figure 2a). For HPB, the density followed a linear decrease with increasing PF content up to 3 wt% NaHCO_3_ (ρ_HPB_ = 1.06 ± 0.03 g·cm^−3^, ρ_HPB/NaHCO_3_-1_ = 0.96 ± 0.04 g·cm^−3^ and ρ_HPB/NaHCO_3_-3_ = 0.66 ± 0.04 g·cm^−3^). Macroscopically, the same trend toward a more porous structure for increasing PFs was observed (Figure 2c,e,g). The addition of 1 wt% NaHCO_3_ resulted in few, distinctly separated and mainly closed pores (Figure 2e), whereas HPB/NaHCO_3_-3 formed a dense porous network (Figure 2g). Interestingly, a further increase in PF concentration to 5 wt% resulted in an insignificant increase in density (ρ_HPB/NaHCO_3_-5_ = 0.74 ± 0.04 g·cm^−3^; *p* > 0.05) and similar macroscopic appearance compared to HPB/NaHCO_3_-3 (Figure 2g versus Figure 2i).

In contrast to HPB, the density of HPL does not show a linear decrease with increasing PF content, but rather an abrupt density reduction from 1.19 ± 0.03 to 0.77 ± 0.03 g·cm^−3^ at a PF concentration of 1 wt% (*p* << 0.05). At this low PF concentration, the extrudate already showed a dense porous network (Figure 2f). Higher PF concentrations did not lead to a significant change in density (ρ_HPL/NaHCO_3_-3_ = 0.72 ± 0.07 g·cm^−3^ and ρ_HPL/NaHCO_3_-5_ = 0.82 ± 0.04 g·cm^−3^, *p* > 0.05 comparing HPL/NaHCO_3_-1 with HPL/NaHCO_3_-3 and with HPL/NaHCO_3_-5).

All apparent density values are in accordance with the literature, as porous systems produced by chemical foaming can achieve a maximum density decrease of approximately 50%, resulting in so-called high-density foams [69]. In detail, similar density reductions have been reported for porous TPU systems manufactured via injection molding after the addition of 3 wt% of endo- and exothermic PFs (40% reduction, compared to 37% for HPB/NaHCO_3_-3 and 39% for HPL/NaHCO_3_-3, Figure 2a) [85]. In another study, 2 wt% of NaHCO_3_ in PLA-based formulations prepared by HME resulted in a density reduction of 20–35% [71]. If a density decrease above 50% is desired other foaming methods such as the addition of supercritical gases (CO_2_ or N_2_) and particulate leaching should be used [65].

The fact that the density appears to reach a minimum despite further addition of PF can be attributed on the one hand to limited amounts of gas generated by such chemical PFs and on the other hand to the so-called “gas containment limit” phenomenon [69,87,88]. The latter describes that above a certain PF concentration, the pores tend to become smaller due to geometrical constraints (limited volume in the polymer for the increasing amount of gas to develop [69]). As a result, the melt can coalesce or the pores can partially collapse, increasing the overall density [88]. This phenomenon was also found for both TPUs, HPB and HPL. After exiting the die, the formed pores in the HPB/NaHCO_3_-3 and the HPL/NaHCO_3_-1 extrudates increased in size until the polymer was completely solidified. As a result, the pores remained stable, but rather large (see Section 3.4). Therefore, the whole extrudate expanded anisotropically, which was consistent with the increase in ovality (1.46 ± 0.21 mm and 0.56 ± 0.06 mm, Figure 2b,g) compared to all other investigated samples. At elevated PF concentration (i.e., 5 wt% for HPB and 3 and 5 wt% for HPL respectively) more pores expanded faster. As a result of the constant tendency of the pores to grow while being geometrically hindered by the high number of adjacent pores, the pores cannot continue to expand, because some of them partially collapse before the polymer solidifies [69]. As a result, the pores tended to be smaller (see Section 3.4), the extrudates were more compact (Figure 2i,j) and the density slightly increased compared to that of HPB/NaHCO_3_-3 and HPL/NaHCO_3_-1. The ovality of HPB/NaHCO_3_-5 (0.57 ± 0.10 mm) compared to that of HPB/NaHCO_3_-3 (1.46 ± 0.21 mm) significantly decreased (*p* << 0.05). The same was observed for the ovality of HPL/NaHCO_3_-5 (0.16 ± 0.03 mm, Figure 2b) compared to that of HPL/NaHCO_3_-1 (0.56 ± 0.06 mm) and HPL/NaHCO_3_-3 (0.27 ± 0.06 mm). This decrease in ovality to a level corresponding to that of pure HPB (0.62 ± 0.17 mm) and HPL (0.12 ± 0.07 mm) indicates an improved mixing of the melt with the produced gas, resulting in more homogeneous extrudates. Particularly for diffusion-controlled drug delivery systems such as IDDS, homogeneous extrudates with well-defined geometries are essential to guarantee predictable drug-release kinetics [32,89].

The difference in the amount of PF needed to reach the density plateau (3 wt% for HPB and 1 wt% for HPL) was caused by the difference in the hydrophobicity of the soft segments of the two TPU types. Due to its hydrophilic soft segments (i.e., poly(ethylene oxide)), HPL shows a better miscibility with the water vapor, which was generated as gas during the decomposition of NaHCO_3_, than the hydrophobic soft segments of HPB (i.e., (poly)tetrahydrofurane). This resulted in a faster and more homogeneous gas distribution inside the matrix (Figure 2f). In contrast to physical foaming of TPUs via CO_2_, in which the content of hard segments [82] and the soft segment chain length [90] are the main influencing factors in terms of the expansion ratio, the present findings show that the similarity of the gases produced (in the present case H_2_O and CO_2_) together with the TPU type (hydrophobic or hydrophilic) can play a major role in determining the nucleation rate of the pores. This finding is of paramount importance for pharmaceutical applications since both porosity and hydrophobicity have a significant influence on liquid absorption (see Section 3.6) and thus on the drug release [91,92].

### 3.3. True and Skeletal Density

**Figure 3:**
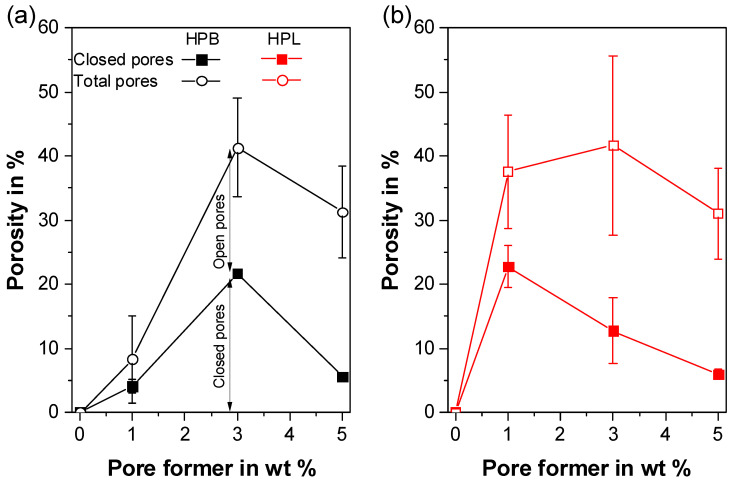
Total porosity and porosities derived from open and closed-cell pores respectively for all formulations comprising (**a**) hydrophobic (HPB) and (**b**) hydrophilic thermoplastic polyurethane (HPL) with and without NaHCO_3_ as pore former. The lines serve for optical guidance only.

The structure of porous thermoplastic foams is usually divided into closed- and open-cell type [69,93]. Closed-cell systems comprise pores that are separated by the polymer matrix. In open-cell systems each pore is connected to one or more adjacent pores providing a passage to the surface of the system. The proportion of open and closed pores, which is an essential parameter for understanding the evolution of the porous structure of foams, was determined by helium pycnometry (Figure 3). The helium gas can only access and fill the open-cell pores. Thereby, the porosity deriving from the pycnometer corresponds to the closed-cell pores, where helium cannot penetrate.

The total porosities presented in Figure 3 refer to the density reduction compared to the density of pure TPU, since the apparent density measurements do not distinguish between the closed- and open-cell volume. The trends of the total porosities show the same inverse trend as the densities (Figure 2). For example, HPB/NaHCO_3_-3 and HPL/NaHCO_3_-3 resulted in the highest overall porosities (41.3% ± 7.7% and 41.6% ± 13.9%, respectively), as the observed densities were the lowest. These porosity values are similar to literature values reported for biodegradable PU foams (25–36%, [66]).

Apart from HPB/NaHCO_3_-1, which had the lowest overall porosity (8.3% ± 6.8%), all investigated samples followed a trend toward decreasing porosity derived from closed-cell pores with increasing PF content. For HPB/NaHCO_3_-1 and HPB/NaHCO_3_-3 the closed-cell pores accounted for 50% of the overall porosity, which dropped to 18% for HPB/NaHCO_3_-5. For HPL/NaHCO_3_-1, the porosity derived from closed-cell pores accounted for 60% of the overall porosity, whereas for HPL/NaHCO_3_-3 and HPL/NaHCO_3_-5 it decreased to 30% and 20%, respectively. This trend indicates that the porous network in both polymers became more interconnected over the whole cross-section of the extrudate, which transformed toward an open-cell structure with increasing PF concentration. Due to an increased amount of gas generated at higher PF concentrations, the number of gas bubbles per unit volume of the polymer increased, causing the porous network to expand towards the surface of the polymer [69]. Finally, at 5 wt% PF both polymers showed very similar results in terms of porosity derived from closed- and open-cell pores verifying a stable process and a homogeneous product.

The importance of investigating the open/closed pores ratio must be emphasized, because it impacts the performance of the two TPUs used as IDDS in a very different manner. For HPB it is expected that the closed pores only influence the mechanical properties of the final formulation, but barely the drug release kinetics, since the closed-cell volume is not accessible to body liquids. On the contrary, for HPL, both open- and closed-cell pores are expected, which will alter the drug release kinetics as both types of pores will be accessible to body liquids due to the highly hydrophilic nature of the polymer. Based on the presented formulations, a wide range of open/closed-cell pore ratios are therefore available, which allows different release behavior of the active ingredient to be achieved.

### 3.4. Pore Morphology

**Figure 4:**
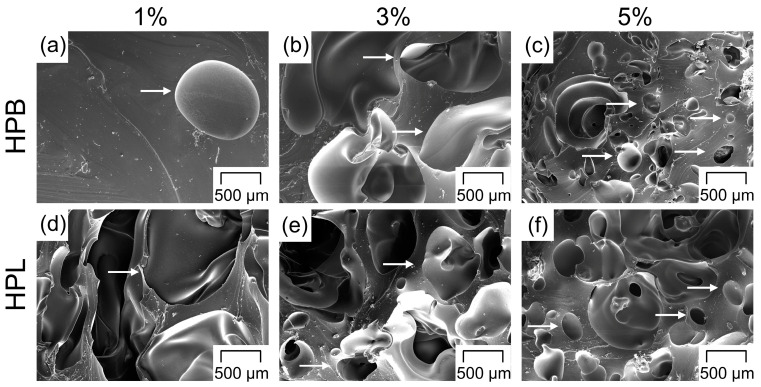
Scanning electron microscopy (SEM) images of the cryofractured surface of the extrudates as a function of the pore former concentration for (**a**–**c**) hydrophobic (HPB) and (**d**–**f**) hydrophilic thermoplastic polyurethane (HPL). The white arrows mark the introduced pores.

For both TPUs, the pore morphology seems to follow a trend towards decreasing pore size and increasing number of pores at higher PF concentrations (Figure 4). Similar to previous investigations, a rather irregular pore shape is found in foamed systems produced via HME [81,94,95] and injection molding [94,96]. As suggested in Section 3.3, for higher PF concentrations, interconnected open pores were found, which can be seen in Figure 4c,f, in which larger pores revealed small-scaled connections to adjacent pores. To understand differences in the pore size homogeneity between the investigated formulations, semiquantitative optical analyses were discussed in the following.

Although mercury intrusion porosimetry is regularly implemented to characterize macroporous materials, the measurement of flexible polymers such as TPUs can often lead to incorrect pore size distributions due to the sample compression caused by the mercury penetration [91,97]. Therefore, microscopy and image analysis were used to characterize the pores in the extrudates, in a similar way as in Ref. [98]. The pore surface area and the average circle equivalent pore diameter (D_a_), both measured by image analysis, were chosen as the descriptor of the pore size characteristics, since none of the investigated formulations revealed regular cubic or spherical pore shapes (Figure 4). According to the Wicksell’s corpuscle problem, the real pore area is on average larger than the observed areas [99]. Hence, the results should only serve as a semiquantitative analysis to highlight the differences in pore size between the formulations.

**Figure 5:**
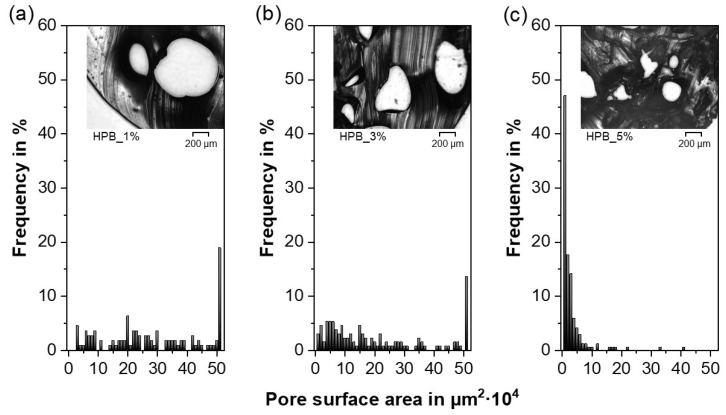
Pore surface area distribution and a representative cross-sectional micrograph for the formulations comprising hydrophobic thermoplastic polyurethane (HPB) and (**a**) 1%, (**b**) 3% and (**c**) 5% NaHCO_3_.

**Figure 6:**
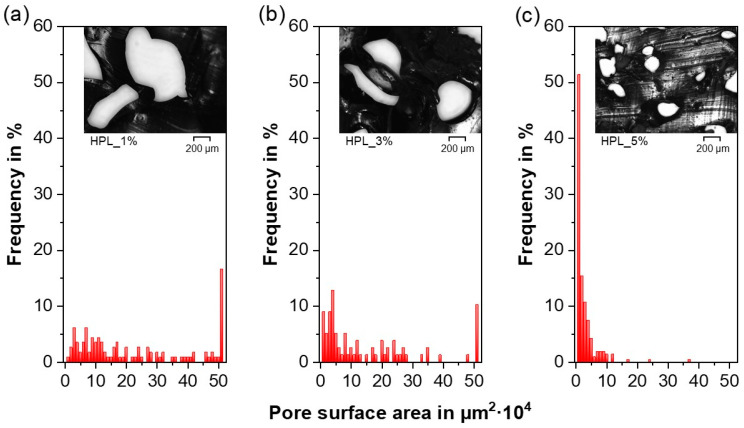
Pore surface area distribution and a representative cross-sectional micrograph for the formulations comprising hydrophilic thermoplastic polyurethane (HPL) and (**a**) 1%, (**b**) 3% and (**c**) 5% NaHCO_3_.

Besides the irregular pore shape, the previously postulated trend towards a decreasing pore size with increasing PF concentrations could be confirmed for both TPUs. The pore surface areas (Figure 5 and Figure 6) and the D_a_ (Table 2) decreased although the overall porosity/density remained constant above a specific PF concentration (≥3 wt% for HPB and ≥1 wt% for HPL, see Section 3.2 and Section 3.3). This trend is related to the “gas containment limit” phenomenon described in Section 3.2, which can cause melt coalescence/collapsing, and lead to the so-called “pizza-sharing situation” [69]. The latter describes that with increasing PF concentration less area is available for the gas to develop inside the polymer matrix, which results in a smaller average size of the gas bubbles. Interestingly, the observed pore sizes are in accordance with those reported both for PU scaffolds produced via different manufacturing methods such as salt leeching (250 µm [64]) and gas foaming (60–300 µm [100]) and for systems produced via HME but using Eudragit^®^ as the polymer matrix (200 µm [101]).

Moreover, the pore surface area distribution became narrower with increasing PF concentration, ranging from a heterogeneous pore size for 1 wt% PF content (Figure 5a and Figure 6a) to a more homogeneous small pore size for high PF concentrations (Figure 5c and Figure 6c) [69]. These findings suggest that the pore size could be further reduced and homogenized if higher PF concentrations than 5 wt% were employed.

In contrast to physical foaming of TPU, for which the soft segment content determines the miscibility with CO_2_ and therefore the pore size [81,82], in the present work, no significant difference in the pore size between HPB and HPL could be observed for constant PF concentrations (Table 2). This finding implies that the miscibility of the PF determined only the nucleation rate (Section 3.2), but not the final pore sizes.

### 3.5. Mechanical Properties

**Figure 7:**
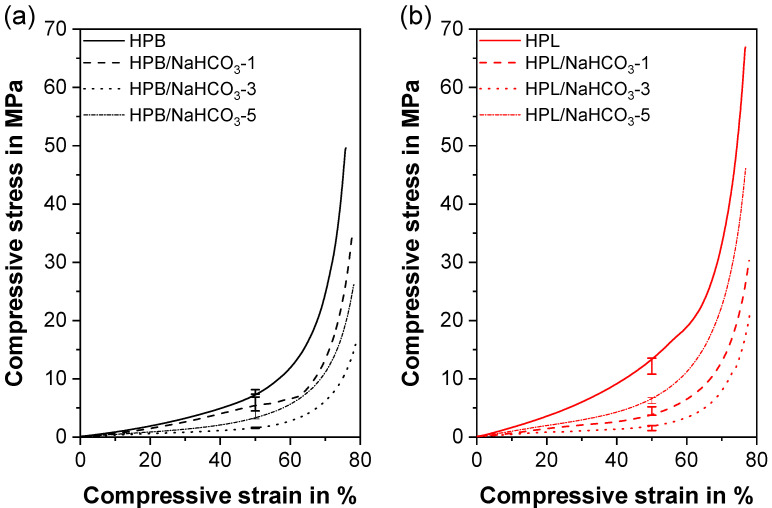
Representative compressive stress–strain curves for the formulations comprising (**a**) hydrophobic (HPB) and (**b**) hydrophilic thermoplastic polyurethane (HPL) with and without NaHCO_3_ as the pore former. The confidence interval for a significance level of 5% for the compressive stress at 50% compressive strain (σ_50_) is marked by error bars.

Under compressive load, the investigated materials underwent the three stage compression behavior typical for conventional elastomeric foams (Figure 7) and TPUs [102]. During stage one, i.e., the initial elastic regime, the cell walls bend and/or stretch, followed by an apparent plateau, where the pores are compressed under large strain, and a second densification increase, where the cell walls impinge upon each other [103], which is often accompanied by buckling of the cell walls. Apart from HPB/NaHCO_3_-1, the first elastic regime was not clearly visible in Figure 7, as the pure materials revealed a very low stiffness. Over the whole strain area, pure HPL (E_C_ = 7.40 ± 0.69 MPa, σ_50_ = 12.16 ± 1.37 MPa, Table 3) had a significantly higher compression modulus (E_C_) and compressive stress at 50% compressive strain (σ_50_) than HPB (E_C_ = 5.34 ± 0.98 MPa, σ_50_ = 7.75 ± 0.37 MPa), since HPL had a higher fraction of hard segments [81,82]. As known from thermoplastic/elastomeric foams [104] and in particular from TPU foams [87,105], the relative density shows a direct correlation with E_C_ and σ_50_. This trend is also valid for the present work. Therefore, the specimens comprising 0% PF had highest E_C_ and σ_50_, while the formulations with the lowest density (HPB/NaHCO_3_-3 and HPL/NaHCO_3_-3) revealed the lowest E_C_ (1.71 ± 0.51 MPa and 1.97 ± 0.79 MPa) and σ_50_ (1.58 ± 0.08 MPa and 1.53 ± 0.42 MPa). However, for HPL, in which all foamed formulations exhibited a comparable density/porosity, HPL/NaHCO_3_-5 had an insignificantly (*p* >> 0.05) higher E_C_ (4.38 ± 1.52 MPa) and significantly (*p* << 0.05) higher σ_50_ (6.26 ± 0.50 MPa) than HPL/NaHCO_3_-1 (3.75 ± 1.25 MPa and 4.48 ± 0.72 MPa). The reason for this finding was twofold. First, the effect of pore size on the compressive behavior cannot be neglected, since both closed and open pores existed simultaneously (Figure 3b). For the same relative density, foams comprising smaller closed pores (HPL/NaHCO_3_-5) exhibited higher E_C_ and σ_50_ values than those with larger pores (HPL/NaHCO_3_-1) [106,107]. This difference may be attributed to different fractions of the solid contained in the cell edges and faces in both foams and the potential influence of enclosed gas [104]. Secondly, besides the mean pore size, also the pore size distribution can influence the compressive properties of cellular structures [106]. Therefore, the much narrower and homogeneous pore size distribution of HPL/NaHCO_3_-5 (137 ± 100 µm) compared to that of HPL/NaHCO_3_-1 (523 ± 281 µm, Figure 6) might be another reason for the different compressive performance particularly in the collapse plateau, despite having the same densities.

In a nutshell, for both TPU types, the formulations containing 1 wt% and 5 wt% PF had comparable compressive properties, which is essential for potential applications as IDDS. At the same time, these formulations had a completely different pore morphology and open/closed pore proportions. Consequently, they offered a broad application spectrum in terms of pharmaceutical performance while ensuring similar mechanical performance.

### 3.6. Liquid Uptake

The liquid uptake of a polymer is of great importance as it directly relates to drug diffusion and drug release [51,91]. Thereby, reproducible and homogeneous pore morphology leads to reproducible liquid uptake profiles and allows one to draw conclusions on the product performance in terms of drug release.

**Figure 8:**
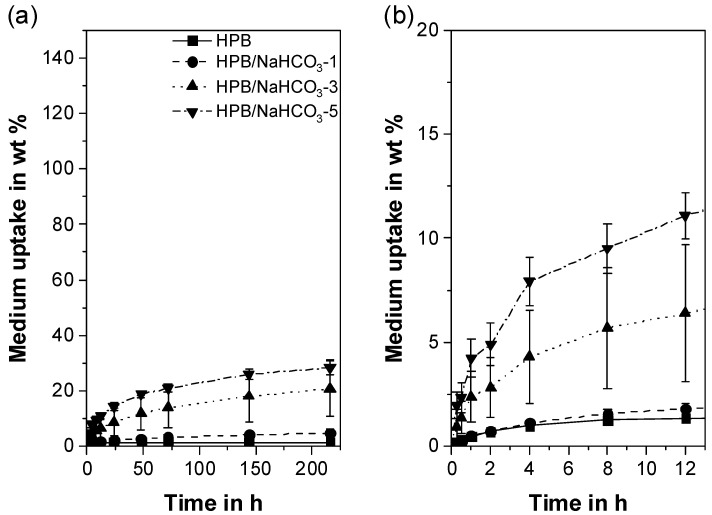
Liquid uptake kinetics for formulations comprising hydrophobic thermoplastic polyurethane (HPB) without and with NaHCO_3_ (**a**) over a period of 9 days (**a**,**b**) a more precise representation of the first 12 h.

For HPB, all formulations show a typical mass increase profile, which is known from the literature for porous systems involving TPUs [108] or other polymers (Figure 8a) [51,91]. After 9 days of incubation, pure HPB shows a mass increase of 1.43 ± 0.07 wt%. This low value is attributed to the weak affinity between the hydrophobic polymer and the hydrophilic medium [44]. The uptake can be increased only by increasing the number of open-cell pores (refer to Section 3.3) through which the liquid can penetrate and be confined [91]. This hypothesis correlates well with our results; the liquid uptake of the porous HPB extrudates shows a direct correlation with the porosity derived from open-cell pores. Due to the small number of open-cell pores, HPB/NaHCO_3_-1 shows a low porosity (Figure 3), and thus a comparable liquid absorption as pure HPB (Figure 8b). After 12 hours, the medium finally diffused through the few open-cell pores, resulting in a significantly higher total mass increase (4.79 ± 1.40 wt%) compared to pure HPB (1.43 ± 0.07 wt%). As expected, an increased number of open-cell pores, as is the case with the formulations HPB/NaHCO_3_-3 and HPB/NaHCO_3_-5 (Figure 3), leads to both a faster and significantly higher liquid uptake (20.8 ± 10.2 wt% and 28.3 ± 2.7 wt% after 9 days, respectively). Overall, the addition of 5 wt% of PF leads to a 20-fold increase in liquid uptake after 9 days of incubation compared to pure HPB. This offers great flexibility in the design of hydrophobic IDDS by simply adjusting the PF concentration.

In addition to the open-cell pores, the width of the pore size distribution and, therefore, the mean pore size diameter influenced the results. This can be discerned from the high standard deviations of the liquid uptake of HPB/NaHCO_3_-3 (Figure 8b) owing to the broad pore size distribution (Figure 5b, Table 2). A large interconnected pore of HPB/NaHCO_3_-3 will confine higher amounts of medium compared to the limited open-cell pores of HPB/NaHCO_3_-1. Although HPB/NaHCO_3_-5 exhibited the smallest and most homogeneous pores (Figure 5c), it still absorbed the highest quantity of medium (Figure 8b). This finding proves that for the hydrophobic TPU used in this study, a high percentage of open-cell pores was more decisive than the pore size.

**Figure 9:**
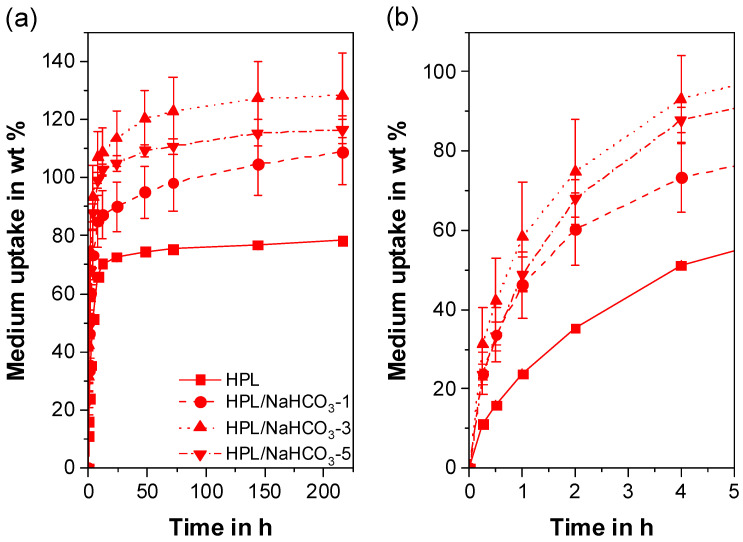
Liquid uptake profiles for formulations comprising hydrophilic thermoplastic polyurethane (HPL) without and with NaHCO_3_ (**a**) over a period of 9 days and (**b**) a more precise representation of the first 5 h.

All formulations comprising HPL (Figure 9a) exhibited a significantly higher and faster liquid uptake than HPB. The liquid uptake of pure HPL reached the plateau already after 24 h at 74.4 ± 0.4 wt%. In contrast to HPB, the liquid uptake of the porous formulations was mainly influenced by the total porosity and not by the number of open-cell pores. This can be explained on the one hand by the fact that the diffusion of the hydrophilic medium into the hydrophilic polymer is promoted and, thus, much faster [58,59]. On the other hand, the liquid absorption can be further increased by a higher total porosity [91]. All porous HPL formulations show a higher liquid absorption than pure HPL. In addition, when the liquid uptake plateau was reached, no significant differences between the porous HPL formulations were observed (*p* >> 0.05; Figure 9b). This finding can be attributed to the similar overall porosities obtained for all three formulations (Figure 3).

Similar to the HPB extrudates, both HPL/NaHCO_3_-1 and HPL/NaHCO_3_-3 revealed rather high standard deviations for the porosity (Figure 3) and a wide pore size distribution. Hence, the standard deviations in the liquid uptake studies were also rather high (13% and 14%, respectively). Same as for HPB/NaHCO_3_-5, due to the more homogenous pore size distribution and smaller average pore size, HPL/NaHCO_3_-5 shows less fluctuations in terms of porosity (Figure 3), a narrow pore surface area distribution (Figure 6) and thus a low relative standard deviation (5%) with regard to liquid uptake. It should be noted that the high deviations in liquid absorption could also be partially caused by the measurement setup itself, especially in the case of large-pored samples (i.e., HPL/NaHCO_3_-1 and HPL/NaHCO_3_-3). These pores may not have adequately trapped the water when the sample was wiped with a paper towel, so that some of the water was removed.

Based on the liquid uptake results, a broad drug-release portfolio can be expected. Diverse formulation possibilities can be considered based on mainly two factors. Firstly, the physicochemical properties of the API in question, which will affect the polymer/API interactions and consequently the drug release [32,40,47]. Secondly, the type of TPU matrix in terms of hydrophobicity. This will define whether only the open-cell pores influence the liquid uptake and consequently the contact surface area for the medium (HPB) [51] or both open- and closed-cell pores impact the liquid uptake capacity of the matrix (HPL). These aspects will be the topic of subsequent studies

## 4. Conclusions

In this study, a one-step HME process was implemented to create porous TPUs intended for IDDS application. A hydrophobic and a hydrophilic TPU were combined with different PF types and concentrations and their influence on the density/porosity, pore morphology, compressive behavior and liquid absorption was studied. Based on the results, the following conclusions were drawn:

- For the TPUs investigated, only PF with decomposition temperatures that overlap with the HME temperature, i.e., NaHCO_3_, led to a reproducible porous network.
- For both TPU types and the employed experimental setup, the porosity increased with increasing PF concentrations and reached a maximum at 40%. However, the appropriate PF concentration to reach the porosity maximum depended on the TPU type (i.e., hydrophobic or hydrophilic), as the gases produced due to PF decomposition during HME played a major role in the pore nucleation kinetics, leading to faster pore formation for the hydrophilic TPU. The final pore size was not affected by the hydrophilicity of the TPUs, but rather by geometrical constraints. As a result, the average pore size decreased and the pore size distribution became narrower with increasing PF content. In addition, the porous network became more interconnected whereby the initially closed-cell pores gradually rearranged into open-cell pores with increasing PF concentration.
- The compression properties of the porous extrudates were strongly influenced by the porosity and the pore morphology. Due to the smaller pores, extrudates comprising 5 wt% of PF resulted in a comparable compression modulus and compressive stress as extrudates containing 1 wt%, despite their difference in porosity. Consequently, IDDS with a broad application portfolio in terms of porosity, but comparable and consistent mechanical properties were produced.
- Depending on the hydrophilicity of the TPU, a wide range of liquid uptake was obtained, which correlated directly with the extrudate porosity. For the hydrophobic TPU, a 20-fold increase in mass was found, since liquid uptake was determined by the number of open-cell pores. Due to the high liquid uptake of the pure hydrophilic TPU, only a 1.6-fold mass increase, which was dependent on the total porosity, was achieved for the porous hydrophilic extrudates.
- Based on the results of the extrudate characterization, both hydrophobic and hydrophilic drugs can be considered as candidates. The release of a hydrophobic API will be increased due to the larger surface area provided by the open-cell pores of HPB and by the higher total porosity of HPL. However, due to the rapid liquid uptake of the porous HPL extrudates, PF concentrations lower than 1 wt% should be investigated to reduce the uptake of large medium amounts and avoid the immediate release of the API. Due to its high solubility in aqueous media, a hydrophilic API might be mainly considered in combination with the porous HPB carrier for a delayed, extended-release formulation.

In summary, these results provide a deeper understanding of porous TPU-based carriers continuously produced via HME. The proposed foaming strategy is not only beneficial for the development of the next generation IDDS, but also promising for a wide range of drug delivery systems.

## 5. Patents

A patent application was filed as a result of this work (registration date: 09.04.2020, file number: 10 2020 110 089.2).

## Figures and Tables

**Table 1:** Final formulations comprising hydrophobic TPU (HPB), hydrophilic TPU (HPL) and NaHCO_3_ as the spore former.

Sample Designation	Polymer Type	NaHCO_3_ (wt%)
HPB	Hydrophobic TPU	-
HPB/NaHCO_3_-1		1
HPB/NaHCO_3_-3		3
HPB/NaHCO_3_-5		5
HPL	Hydrophilic TPU	-
HPL/NaHCO_3_-1		1
HPL/NaHCO_3_-3		3
HPL/NaHCO_3_-5		5

**Table 2:** Average circle equivalent pore diameter (D_a_) of the porous extrudates comprising hydrophobic (HPB), hydrophilic thermoplastic polyurethane (HPL) and NaHCO_3_ as the pore former.

Sample Designation	D_a_ (µm)
HPB/NaHCO_3_-1	596 ± 240
HPB/NaHCO_3_-3	475 ± 247
HPB/NaHCO_3_-5	146 ± 108
HPL/NaHCO_3_-1	523 ± 281
HPL/NaHCO_3_-3	405 ± 290
HPL/NaHCO_3_-5	137 ± 100

**Table 3:** The mean and confidence interval for a significance level of 5% for the compression modulus (E_C_) and compressive stress at 50% compressive strain (σ_50_) for all investigated formulations comprising hydrophobic (HPB) and hydrophilic (HPL) thermoplastic polyurethanes.

Sample Designation	E_C_ (MPa)	σ_50_ (MPa)
HPB	5.34 ± 0.98	7.75 ± 0.37
HPB/NaHCO_3_-1	2.51 ± 0.68	5.67 ± 1.19
HPB/NaHCO_3_-3	1.71 ± 0.51	1.58 ± 0.08
HPB/NaHCO_3_-5	2.41 ± 0.65	3.76 ± 0.65
HPL	7.40 ± 0.69	12.16 ± 1.37
HPL/NaHCO_3_-1	3.75 ± 1.25	4.48 ± 0.72
HPL/NaHCO_3_-3	1.97 ± 0.79	1.53 ± 0.42
HPL/NaHCO_3_-5	4.38 ± 1.52	6.26 ± 0.50

## Supplementary Materials

The following are available online at https://www.mdpi.com/2073-4360/12/12/2950/s1, Figure S1: (a,c) macroscopic images of formulations comprising hydrophobic (HPB) and (b,d) hydrophilic thermoplastic polyurethane (HPL).
