# Supplementary material for: Development of Porous Polyurethane Implants Manufactured via Hot-Melt Extrusion
Source: Polymers (Basel). 2020 Dec 10;12(12):2950. doi: 10.3390/polym12122950 (PMC7764633; doi:10.3390/polym12122950)
Supplement: Supplementary file 1 [file polymers-12-02950-s001.pdf]

Development of porous polyurethane implants manufactured via hot-melt extrusion

Ioannis Koutsamanis [1,2,+], Martin Spoerk [1,+], Florian Arbeiter [3], Simone Eder [1,*] and Eva Roblegg [1,2,*]

[1] Research Center Pharmaceutical Engineering GmbH, Inffeldgasse 13, 8010 Graz, Austria; ioannis.koutsamanis@rcpe.ar (I.K.); martin.spoerk@rcpe.at (M.Sp.); simone.eder@rcpe.at (S.E.)
[2] Institute of Pharmaceutical Sciences, Pharmaceutical Technology and Biopharmacy, University of Graz, Universitaetsplatz 1, 8010 Graz, Austria; eva.roblegg@uni-graz.at (E.R.)
[3] Institute of Materials Science and Testing of Polymers, Montanuniversitaet Leoben, Otto Gloeckel-Straße 2, 8700 Leoben, Austria; Florian.Arbeiter@unileoben.ac.at (F.A.)
+Both authors contributed equally to this work
* Correspondence: eva.roblegg@uni-graz.at (E.R.); Tel: +43 (0) 316 380 8888, Fax: +43 (0)316 380 – 9100

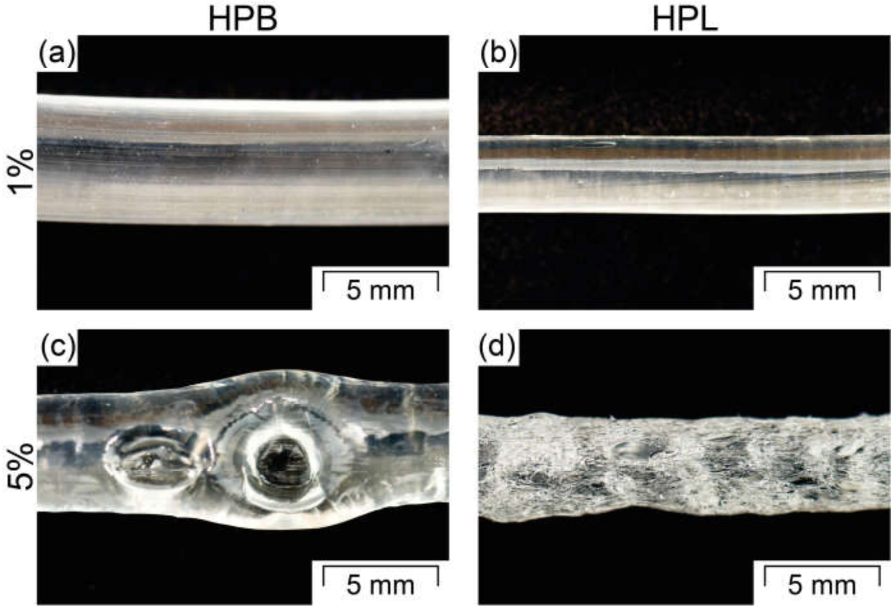

Figure S1. (a, c) macroscopic images of formulations comprising hydrophobic (HPB) and (b, d) hydrophilic thermoplastic polyurethane (HPL).
